# Comparative Transcriptome Analysis Revealed Genes Regulated by Histone Acetylation and Genes Related to Sex Hormone Biosynthesis in *Phytophthora infestans*

**DOI:** 10.3389/fgene.2020.00508

**Published:** 2020-05-21

**Authors:** Xiao-Wen Wang, Jia-Lu Lv, Ya-Ru Shi, Li-Yun Guo

**Affiliations:** Ministry of Agriculture (MOA) Key Lab of Pest Monitoring and Green Management, College of Plant Protection, China Agricultural University, Beijing, China

**Keywords:** oomycetes, epigenetic mechanisms, oospores, α hormones, P450, transcription factors, RNA-seq

## Abstract

Late blight caused by *Phytophthora infestans*, is one of the most devastating diseases of potato, and was responsible for the death of millions of people during the Irish Potato Famine in the nineteenth century. *Phytophthora infestans* is a heterothallic oomycete that typically requires two compatible types (mating types), A1 and A2, to complete sexual reproduction (i.e., oospore production). Oospores have critical effects on disease epidemiology because they serve as the primary inoculum in subsequent growing seasons. The sexual reproduction of *Phytophthora* species is regulated by α hormones. In previous studies, we proved that transformants in which selected histone deacetylase (*HDAC*) genes are silenced exhibit abnormal hormone production. In the current study, we compared the transcriptomes of *HDAC*-silenced and wild-type strains to explore the genes regulated by HDAC and the genes involved in sex hormone biosynthesis in *Phytophthora* species. A total of 14,423 transcripts of unigenes were identified in the wild-type strain, the *HDAC* family-silenced transformant (HDST), and the *HDAC7*-silenced transformant (H7ST). After comparing the intergroup gene expression levels, 1,612 unigenes were identified as differentially expressed among these strains. The expression levels of 16 differentially expressed genes (DEGs) were validated by quantitative real-time PCR. The functional annotation of the DEGs by gene ontology and Kyoto Encyclopedia of Genes and Genomes pathway analyses indicated that HDACs affect the expression of genes related to metabolic and biosynthetic processes, RNA processing, translation, ribosome biogenesis, cellular structural constituents, RNA binding, and protein binding. Moreover, HDAC7 specifically influences the transcription of genes associated with transport, methylation, mitochondria, organelle inner membranes, receptors and transporters, and hydrolase activities. We also identified 18 candidate genes related to α hormones biosynthesis, including a gene encoding the NF-Y transcription factor (*PITG_10861*). The overexpression of *PITG_10861* increased the production of hormone α2. The results of this study revealed *P. infestans* genes affected by histone acetylation. The data presented herein provide useful inputs for future research on the epigenetic mechanisms and mating behaviors of *Phytophthora* species.

## Introduction

*Phytophthora* species are filamentous organisms that cause severe diseases in many plant species, resulting in substantial economic losses in the global agricultural industry. *Phytophthora* species are oomycetes, which form a specific eukaryotic group in the kingdom Stramenopila, and are phylogenetically close to photosynthetic algae (Sogin and Silberman, 1998; Baldauf et al., 2000; Yoon et al., 2002; Adl et al., 2005). *Phytophthora infestans* is one of the most destructive plant pathogens. It is the causal pathogen of the Irish Potato Famine, which resulted in the death of millions of people in the middle of the nineteenth century. Additionally, it is a model heterothallic *Phytophthora* species, which normally requires two compatible mating types (A1 and A2) producing specific hormones to initiate selfing and hybridizations in cocultures of the same species (Judelson, 1997; Qi et al., 2005; Ko, 2007; Ojika et al., 2011).

Previous studies revealed that the sexual reproduction of *Phytophthora* species is regulated by two interspecies universally active, diterpene hormones, α1 and α2 (Ko, 1980; Qi et al., 2005; Ojika et al., 2011), and requires two distinct and independent processes, hormone production and hormone reception (Ko, 1978). Hormone α1, secreted by A1 strains, induces oospore production in A2 strains, whereas hormone α2, secreted by A2 strains, induces oospore production in A1 strains. The production of oospores in strains of homothallic species is stimulated by self-produced α hormones (α1, α2, or both) (Ko, 1980). Using deuterium-labeled ^1^H, Ojika et al. (2011) proved that A2 strains can use phytol to synthesize α2, and A1 strains can use α2 to synthesize α1. However, the hormone synthesis-related genes remain to be investigated.

Gene expression in eukaryotes can be regulated by epigenetic mechanisms, especially DNA methylation and histone modifications (Hattori and Ushijima, 2014). Histone acetylation occurs at the amino groups of lysine residues at the N-terminus of histone tails, and is regulated by histone acetyltransferases (HATs) and histone deacetylases (HDACs) (Allfrey et al., 1964; Brownell and Allis, 1996; Pflum et al., 2001). The HATs, which act as “writers,” are responsible for transferring an acetyl moiety from acetyl coenzyme A (acetyl CoA) to the ε-amino group of specific lysine residues at the histone N-terminal tails (Bertos et al., 2001; Thiagalingam et al., 2003). Consequently, RNA polymerase and other transcription factor complexes interact with DNA (Hong et al., 1993), leading to up-regulated gene expression (Mukherjee et al., 2012). In contrast, HDACs function as epigenetic “erasers” (i.e., HAT antagonists), which remove acetyl groups from histones, thereby down-regulating gene expression. The balance between “erasers” and “writers” makes chromatin regulation a dynamic process. Many studies have indicated that the expression of biosynthesis-related genes is regulated by histone acetylation. In mammals, the key regulator of cholesterol biosynthesis, sterol-regulatory element binding protein (SREBP)-2, is regulated by sirtuin (Sirt6) (Tao et al., 2013). In the endogenous cholesterol synthesis pathway, a transcription factor, CCAAT-binding factor/nuclear factor Y (CBF/NF-Y), is required for the sterol-regulated transcription of the gene encoding a key regulatory enzyme, 3-hydroxy-3-methylglutaryl-coenzyme A (HMG-CoA) synthase (Dooley et al., 1998). In *Arabidopsis thaliana*, the HDACs SRT1 and SRT2 interact with EIN2 nuclear-associated protein 1 (ENAP1) to regulate the extent of the H3K9 acetylation in the ethylene signaling pathway and mediate transcriptional repression (Zhang et al., 2018). In *Aspergillus flavus*, the epigenetic reader SntB, which is a transcriptional regulator of the sterigmatocystin biosynthetic gene, controls the global levels of H3K9K14 acetylation and secondary metabolite synthesis (Pfannenstiel et al., 2018). The cytoplasmic effector PsAvh23 produced by the soybean pathogen *Phytophthora sojae* modulates HATs in plants, suppresses the activation of host defense genes by disrupting HAT complex functionality during an infection, and ultimately increases the plant susceptibility to disease (Kong et al., 2017). However, the mechanism underlying the metabolic biosynthesis regulated by histone acetylation in oomycetes remains unclear.

We previously analyzed the expression of *HAT* and *HDAC* genes during various *P. infestans* biological stages (Wang et al., 2016). We also generated transformants in which at least one *HDAC* gene was silenced, which altered compatible type. Therefore, we speculated that the biosynthesis of hormones in *P. infestans* might be regulated by histone acetylation. The aim of this study was to investigate the genes regulated by histone acetylation and identify the genes related to sex hormone biosynthesis. We selected the wild-type (WT) *P. infestans* MX5-1 strain and two *HDAC*-silenced transformants for RNA sequencing (RNA-seq) and comparative transcriptome analyses. We then screened for genes related to hormone biosynthesis by comparing the gene expression profiles with the phenotypes of the strains.

## Materials and Methods

### Experimental Design

**FIGURE 1:**
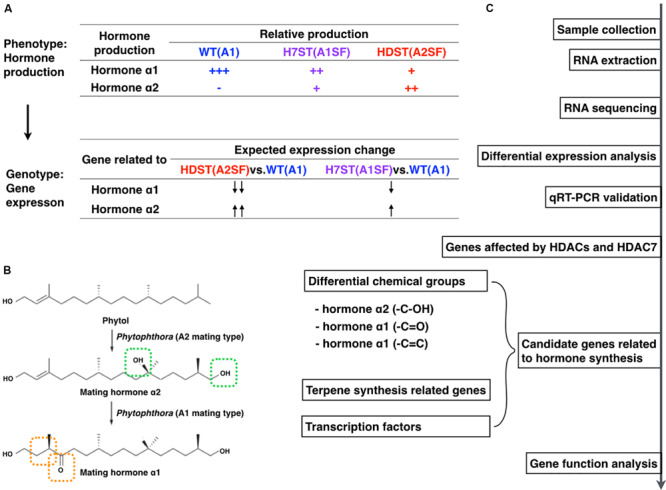
Experimental design and workflow. **(A)** Expected expression-level changes to genes associated with sex hormone biosynthesis in WT, HDST, and H7ST strains according to the differences in hormone production in these strains. **(B)** Chemical structures of α1 and α2 hormones and the predicted hormone biosynthesis pathway with phytol as a precursor (modified from Ojika et al., 2011). **(C)** Study workflow.

The WT A1 strain MX5-1 as well as the *HDAC* family-silenced transformant HDST43 and the *HDAC7*-silenced transformant H7ST20 were analyzed in this study (Supplementary Figure S1). The *HDAC* family-silenced transformants were obtained with vectors containing a partial antisense *PiHDAC7* open reading frame (ORF) sequence that is conserved in *P. infestans HDAC* genes. The *HDAC7*-silenced transformants were generated using vectors containing the 5′ and 3′ untranslated regions of *PiHDAC7*. The down-regulated *HDAC* expression of gene-silenced transformants was confirmed by quantitative real-time (qRT)-PCR analyses (Supplementary Figure S1). The two transformants grew slowly and their mating types differed from that of the WT MX5-1 (Supplementary Figure S2). These three strains have a nearly identical genetic background, but vary regarding mating behavior (phenotypes). Their gene expression profiles were compared based on RNA-seq data, which were validated by qRT-PCR (Figure 1). Furthermore, differential expression profiles were examined to screen for genes potentially associated with sex hormone biosynthesis, including genes related to diverse hormone chemical structures, genes involved in terpene biosynthesis pathways, and genes encoding regulators of sex hormone biosynthesis (e.g., transcription factors). We selected one candidate gene and evaluated its effect on hormone production. All experiments were repeated with different sets of biological samples.

### Sample Collection and RNA Extraction

The HDST43, H7ST20, and WT MX5-1 strains were grown on 290 tomato rye agar at 18^°^C in darkness for 6 days (Guo et al., 2010). All strains were analyzed with three biological replicates, each comprising five plates (60 mm). The mycelia were collected and ground in liquid nitrogen, after which total RNA was extracted with the NucleoSpin RNA Plant kit (MACHEREY-NAGEL, Düren, Germany). The purity and quality of the RNA were assessed with the Thermo NanoDrop 2000 spectrophotometer (Wilmington, United States) and by 1% agarose gel electrophoresis.

### Transcriptome Profiling

After completing the RNA quality control procedures, mRNA was enriched with oligo(dT) beads and fragmented randomly in fragmentation buffer before first-strand cDNA was synthesized with random hexamers and reverse transcriptase. A custom second-strand synthesis buffer (Illumina, San Diego, CA, United States) was added with dNTPs, RNase H, and *Escherichia coli* polymerase I to generate the second strand via nick translation. The final cDNA library was prepared after a round of purification, terminal repair, A-tailing, ligation of sequencing adapters, size selection, and PCR enrichment. The cDNA library concentration was determined with the Qubit 2.0 fluorometer (Life Technologies), and then diluted to 1 ng/μl before checking the insert size with the Agilent 2100 Bioanalyzer (Agilent, Santa Clara, CA, United States) and by quantitative PCR to ensure accuracy. Libraries were sequenced with the Illumina HiSeq X Ten RNA-seq platform (Illumina, San Diego, CA, United States). The resulting raw data were transformed into sequenced reads by base calling. The raw data were recorded in a FASTQ file, which contained sequence information (reads) and the corresponding sequencing quality details. Each biological replicate was treated as an independent sample. Raw reads were then filtered to remove reads containing adapters or those of low quality. All remaining clean reads were mapped to the *P. infestans* reference genome (the EMBL/Genbank/DDBJ databases under the accession GCA_000142945.1) with the TopHat2 software^1^ (Kim et al., 2013).

### Differential Expression Analysis

Gene expression levels were recorded in terms of transcript abundance, which was measured by counting the reads mapped to genes or exons. The expected number of fragments per kilobase of transcript sequence per million base pairs sequenced (FPKM) was considered for examining the sequencing depth and gene fragment lengths (Trapnell et al., 2010). The HTSeq program was used to analyze the gene expression levels. Specifically, an FPKM value of 1 was set as the threshold for determining whether a gene was expressed (Anders et al., 2015). To compare gene expression levels under different conditions, an FPKM distribution diagram and violin plot was applied. The final FPKM for each strain was the mean value of the three biological replicates. The correlation analysis between samples of each strain was used to test the reliability of the transcriptome profiles. When the correlation coefficient is larger than 0.8, the three biological samples were used as replicates in the analyses of differentially expressed genes (DEGs). The DEGs between each sample pair were completed with the DESeq R package (Robinson et al., 2010). The *p*-values were adjusted based on *q*-values. A *q*-value < 0.05 was set as the threshold for determining significant differences in expression. The DEGs were functionally annotated based on gene ontology (GO) and Kyoto Encyclopedia of Genes and Genomes (KEGG) pathway enrichment analyses. The GO enrichment analysis was completed according to the GO-seq R package-based Wallenius non-central hypergeometric distribution (Young et al., 2010). The manually curated KEGG databases were related to genomes, biological pathways, diseases, drugs, and chemical substances. Pathway enrichment analyses of the DEGs with the KOBAS software identified significantly enriched metabolic or signal transduction pathways (Mao et al., 2005).

### Quantitative Real-Time PCR Validation

We selected some genes to validate the transcriptome data by qRT-PCR, with the elongation factor gene (*PiEF1*) as an internal control. Regarding the cDNA synthesis, 1 μg total RNA was reverse transcribed with the oligo(dT)_18_ primer and Reverse Transcriptase M-MLV (TaKaRa Bio Inc., Shiga, Japan) following the manufacturer’s instructions (Supplementary Table S1). A qRT-PCR assay was performed with the ABI 7500 Real-Time PCR system (Applied Biosystems, Foster City, CA, United States) as previously described (Wang et al., 2016). Gene expression levels were normalized against the expression of *PiEF1*. Each RNA sample was analyzed in triplicate. The experiments were repeated once with a different set of biological samples. The expression data were analyzed with the ABI 7500 system software.

### Plasmid Construction and Generation of *Phytophthora infestans* Transformants

The pTOR-X vector, which is pTORmRFP4 with an added *Xba*I restriction site, was used to construct recombinant plasmids for transforming *P. infestans*. The *PITG_10861* ORF was amplified with specific primers (Supplementary Table S1) and the cDNA of the WT *P. infestans* A2 strain 80787-94L. The *PITG_10861* ORF was inserted into the pTOR-X vector in the sense orientation and was verified by DNA sequencing. The constructed recombinant plasmid was used for overexpressing *PITG_10861*. The 80787-94L strain was transformed as previously described (Judelson et al., 1991; Mcleod et al., 2008; Guo et al., 2017). All transformants were validated by measuring mycelial *PITG_10861* transcript levels in a qRT-PCR assay with specific primers (Supplementary Table S1).

### Detection of α Hormone Production

The bioassay method developed by Ko (1980) was used for analyzing hormone production and reception. Specifically, *P. infestans* strains MX5-1 (A1) and HCl7-7-2 (A2SF) were used as the receptors of the transformant-produced hormones α2 and α1, respectively. To evaluate the hormone production of the transformants, a culture block of the hormone receptor strain (1-day-old) was placed at the center of a Petri plate (60 mm in diameter) and covered with a polycarbonate membrane (Millipore HTTP04700, 0.22 μm, 47 mm in diameter). A transformant block (4-day-old) was placed on top. The cultures were then incubated in a humid chamber in darkness for 3 weeks. For each test, the oospores produced on each block were counted. All experiments were repeated with three replicates per treatment.

## Results

### Differences in the Hormone Production of Three Tested Strains

*Phytophthora* species mating types are determined based on the type of hormone production and reception. The silencing of *HDAC* genes resulted in transformants that differed from the WT strain regarding hormone production. The *HDAC* family-silenced transformant HDST43 was A2SF (self-fertile and produced more oospores with the A1 strain than with the A2 strain), whereas the *HDAC7*-silenced transformant H7ST20 was A1SF (self-fertile and produced more oospores with the A2 strain than with the A1 strain). The two transformants produced both hormones α1 and α2. However, the bioassay data revealed that HDST43 produced more hormone α2, whereas H7ST20 produced more hormone α1 (Supplementary Figure S2).

### Overview of Illumina Sequencing Data

To investigate the effect of histone acetylation on gene expression as well as the molecular mechanism underlying the biosynthesis of sex hormones, we conducted next-generation sequencing of WT, HDST, and H7ST *P. infestans* strains, which varied in terms of sex hormone production. The sequencing data were used for a comparative transcriptomic analysis. After filtering the raw reads, more than 44 million clean reads (>6.5 Gb) were generated for each mycelial sample of these strains. Detailed sequencing results are summarized in Table 1.

**TABLE 1:** Summary statistics of transcriptome sequencing for three different phenotypes of *Phytophthora infestans*.

Sample name	Raw reads	Clean reads	Clean bases	Error rate (%)	Q20 (%)	Q30 (%)	GC content (%)
WT_rep1	55275070	52737882	7.91G	0.03	94.9	87.99	56.41
WT_rep2	57176716	55830466	8.37G	0.02	96.98	92.52	56.3
WT_rep3	54773296	52301750	7.85G	0.03	95.05	88.29	56.65
HDST_rep1	56172994	54633116	8.19G	0.02	96.76	92.07	56.87
HDST_rep2	50812758	48512390	7.28G	0.03	94.99	88.21	57.02
HDST_rep3	48011368	45800452	6.87G	0.03	94.75	87.75	57.01
H7ST_rep1	45672608	44382744	6.66G	0.02	96.69	91.89	57.04
H7ST_rep2	52932310	51465818	7.72G	0.02	96.58	91.66	57.05
H7ST_rep3	52956756	50542662	7.58G	0.03	94.78	87.81	57.23

**FIGURE 2:**
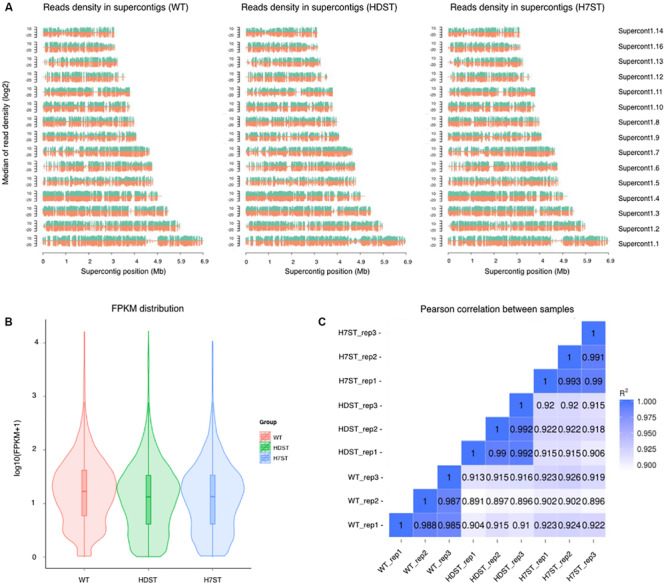
Analysis of the transcriptome data. **(A)** Distribution plot of the mapped reads in supercontigs. The x-axis indicates the chromosome length (in Mb), whereas the y-axis indicates the median log_2_ read density. Green and red indicate the positive and negative strands, respectively. **(B)** Violin plots of the FPKM values for gene expression levels in various samples. The x-axis indicates the sample names, whereas the y-axis indicates the log_10_(FPKM + 1) values. Each violin plot has five statistical magnitudes (maximum value, upper quartile, median, lower quartile, and minimum value). The violin width represents the gene density. **(C)** RNA-seq correlation analysis. Heat maps of the correlation between samples are presented. R^2^, the square of the Pearson correlation coefficient.

The transcriptome assemblies obtained for three strains were pooled and used to assemble full-length transcripts with the *P. infestans* reference genome. After eliminating the redundant transcripts, 72.68–79.13% of the clean reads for these samples were uniquely mapped to the reference genome (Figure 2A and Supplementary Table S2). Furthermore, 91.8–95% of the reads were located within exons (Supplementary Figure S3). The transcriptome sequencing of mycelia identified a total of 27,657 unigenes (Supplementary Table S3). In this study, a gene was considered to be expressed in a strain if its transcript was detected in the cDNA library for all biological replicates. A total of 14,423 transcripts of unigenes were detected in the WT, HDST, and H7ST strains, but relatively few genes were highly expressed (Figure 2B and Supplementary Table S3). For each strain, the data were highly correlated among three biological replicates (Pearson’s *r* > 0.98) (Figure 2C), suggesting the transcriptome profiles were highly reproducible and reliable.

### Functional Enrichment Analysis of Differentially Expressed Genes

**FIGURE 3:**
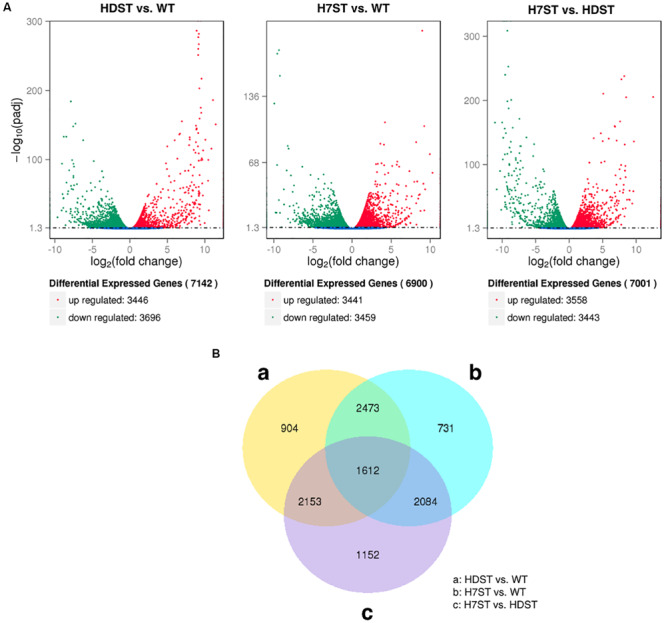
Differentially expressed genes (DEGs) in three phenotypically diverse strains. **(A)** Volcano plot for DEGs. The x-axis indicates the gene expression fold-changes between different strains, whereas the y-axis indicates the significance of the differences. Significantly up- and down-regulated genes are highlighted in red and green, respectively. The blue dots indicate the genes that were not differentially expressed between strains. **(B)** Venn diagram of DEGs. The sum of the numbers in each circle represents the total number of expressed genes within a comparison, whereas the numbers in the overlapping areas represent the number of expressed genes shared between groups.

To investigate the effect of histone acetylation on gene expression in *P. infestans*, we compared the three transcriptomes. We identified 7,142 DEGs between the HDST and WT strains, of which 3,446 and 3,696 DEGs exhibited up- and down-regulated expression, respectively. We also identified 6,900 DEGs between the H7ST and WT strains, including 3,441 and 3,459 DEGs with up- and down-regulated expression, respectively. Additionally, 7,001 DEGs were detected between the H7ST and HDST strains, of which 3,558 and 3,443 DEGs exhibited up- and down-regulated expression, respectively (Figure 3A). An exploration of the global transcriptional changes among samples revealed the co-expression of 4,085 DEGs between the HDST vs. WT and H7ST vs. WT comparisons as well as 1,612 DEGs among the HDST vs. WT, H7ST vs. WT, and H7ST vs. HDST comparisons (Figure 3B).

**FIGURE 4:**
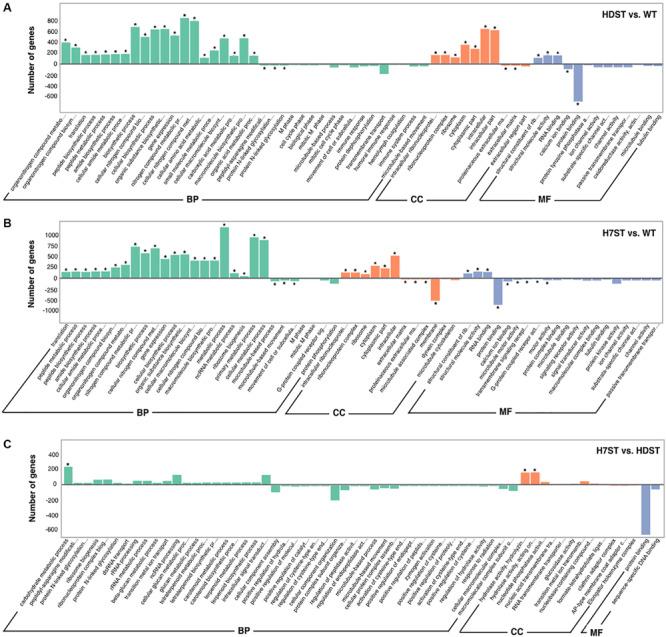
Gene ontology functional classification of differentially expressed genes. The x-axis indicates the enriched GO terms, whereas the y-axis indicates the number of differentially expressed genes in the HDST vs. WT **(A)**, H7ST vs. WT **(B)**, and H7ST vs. HDST **(C)** comparisons. Different colors are used to distinguish the biological process, cellular component, and molecular function GO categories. The enriched GO terms are marked with an asterisk. The ± numbers represent the up-/down-regulated genes.

To functionally characterize the DEGs, we performed a GO enrichment analysis. The 10 most significantly overrepresented GO terms were as follows: organonitrogen compound metabolic and biosynthetic process, translation, peptide metabolic and biosynthetic process, gene expression, intracellular ribonucleoprotein complex, ribosome, cytoplasm, structural constituent of ribosome, RNA binding, and protein binding (Figure 4 and Supplementary Table S4). The cellular metabolic and biosynthetic processes were the two largest subcategories within the biological process category. The most abundant cellular component subcategories were ribosome and intracellular, whereas the two largest molecular function subcategories were binding and catalytic activity. The GO analysis of the *HDAC*-silenced transformants and the WT strain implied that HDACs affect the expression of diverse genes, including those related to metabolic and biosynthetic processes, RNA processing, translation, ribosome biogenesis, ribosomal structural constituents, intracellular and extracellular components, the cytoplasm and organelles, RNA binding, protein binding, ion binding, and nucleic acid binding. We previously identified eight Class I and II HDACs in the *P. infestans* genome, with *HDAC7* belonging to Class II (Wang et al., 2016). A comparison of the DEGs between H7ST vs. WT and H7ST vs. HDST indicated that HDAC7 specifically influences the expression of genes associated with transport, methylation, mitochondria, organelle inner membranes, receptors, transporters, and hydrolase activity.

The functions of the DEGs were also examined based on a KEGG pathway enrichment analysis. The DEGs detected in the HDST vs. WT, H7ST vs. WT, and H7ST vs. HDST comparisons were related to 25 pathways (Supplementary Table S5). Most of the DEGs between the HDST and WT strains appear to contribute to ribosome biogenesis, metabolic pathways, and RNA transport. The genes associated with fatty acid degradation were expressed at lower levels in the HDST strain than in the WT strain. In contrast, the expression levels of some of the DEGs involved in the biosynthesis of amino acids were up-regulated in the HDST strain. Similar trends were observed for the DEGs between the H7ST and WT strains. Additionally, some of the DEGs with lower expression levels in the H7ST strain than in the WT strain were related to endocytosis, regulation of autophagy, and tyrosine metabolism. Moreover, most of the DEGs between the H7ST and HDST strains were significantly associated with cyanoamino acid metabolism, starch and sucrose metabolism, ribosome biogenesis, and fatty acid degradation. The expression levels of DEGs involved in nicotinate and nicotinamide metabolism were down-regulated in the H7ST strain relative to the corresponding expression in the HDST strain, whereas genes related to DNA replication and other glycan degradation exhibited the opposite trend.

### Validating the Differentially Expressed Genes Identified With RNA-Seq Data by Quantitative Real-Time PCR

**FIGURE 5:**
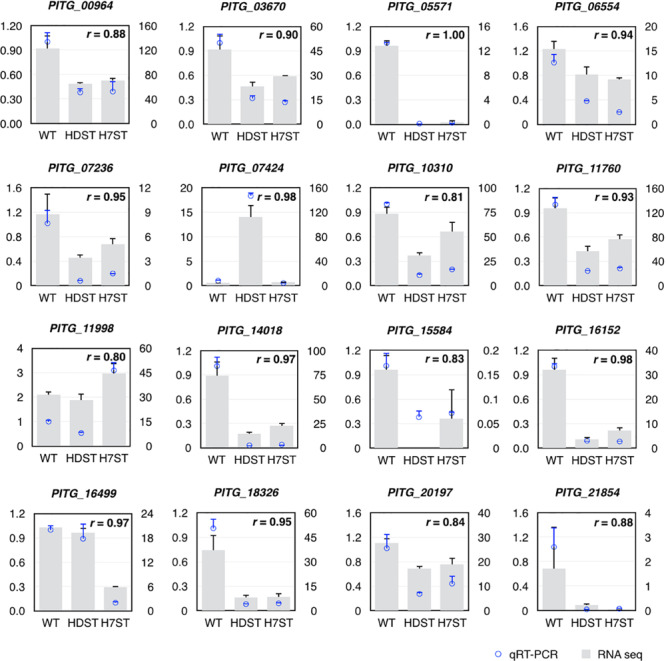
Validation of differentially expressed genes by qRT-PCR. The left y-axis indicates the gene expression fold-changes based on the qRT-PCR data, whereas the right y-axis indicates the FPKM values based on the RNA-seq data, which represent the relative expression of the same gene in the HDST, H7ST, and WT strains. The *r* value represents the Pearson correlation coefficient of two data sets.

To assess the reliability of the DEGs identified by the comparative transcriptomic analysis, we randomly selected 16 genes that were differentially expressed among strains (fold-change > 2) and expressed in at least one sample (FPKM > 1) for a qRT-PCR assay. The qRT-PCR data were highly consistent with the RNA-seq results (Pearson’s *r* > 0.80, *p* < 0.01; Figure 5).

### Expression of Genes Related to Terpene Biosynthesis

In *Phytophthora* species, hormones α1 and α2 are straight-chain diterpenes. Because the terpene pathway in *Phytophthora* species has not been characterized, we screened for genes related to terpene biosynthesis and generated putative pathways according to the KEGG pathway enrichment analysis (Supplementary Figure S4). There are two main terpenoid backbone biosynthesis pathways, namely the mevalonate (MAV) pathway and the 2-C-methyl-D-erythritol 4-phosphate (MEP) pathway. After the terpenoid backbone is synthesized, there are many candidate pathways for diterpene biosynthesis. We identified some *P. infestans* genes related to the MAV pathway, but did not find any orthologs related to the MEP pathway. Moreover, we did not detect any orthologs of genes related to various diterpene biosynthesis pathways predicted in *Phytophthora* species. These results suggested that *Phytophthora* species either have a unique diterpene biosynthesis pathway or do not have one at all.

**FIGURE 6:**
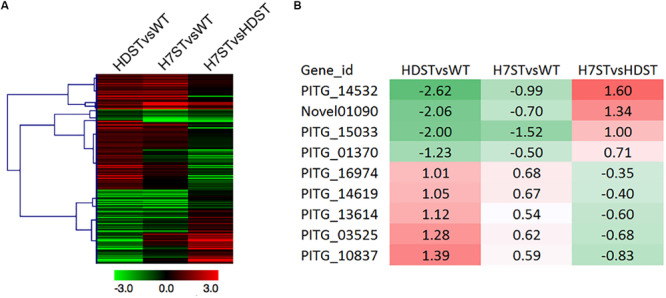
Expression of the genes related to terpene biosynthesis. **(A)** Heat map presenting the expression of 210 genes related to terpene biosynthesis. **(B)** Heat map presenting the expression of nine candidate genes associated with α hormone biosynthesis. Red and green denote genes with up- and down-regulated expression levels, respectively. The numbers represent the log_2_(fold-change) values.

To investigate the terpene biosynthesis-related genes, a GO enrichment analysis was performed, resulting in the identification of 210 candidate genes annotated with 31 GO terms (Figure 6A and Supplementary Table S6). A comparison between the candidate gene expression patterns and the changes in hormone production in the analyzed strains uncovered nine genes potentially associated with hormone production. Among these genes, the following four with down-regulated expression levels in the HDST and H7ST strains might be related to hormone α1 synthesis: *PITG_14532*, *Novel01090*, *PITG_15033*, and *PITG_01370* (*p* < 0.05). The other five genes (*PITG_16974*, *PITG_14619*, *PITG_13614*, *PITG_03525*, and *PITG_10837*), whose expression levels were up-regulated in the HDST and H7ST strains (*p* < 0.05; Figure 6B), might be related to hormone α2 synthesis. The molecular functions of these down-regulated genes are associated with binding during various biosynthesis-related processes, including flavin adenine dinucleotide binding, ion binding, small molecule binding, and nucleic acid binding. The up-regulated gene *PITG_16974* encodes a coenzyme in cofactor metabolic processes. Two other genes with up-regulated expression, *PITG_14619* and *PITG_10837*, encode transferases related to hexosyl groups. The proteins encoded by the remaining two up-regulated genes, *PITG_13614* and *PITG_03525*, have hydrolase activities affecting glycosyl and ester bonds, respectively.

### Expression of Genes Related to Chemical Groups on Hormones Structures

*Phytophthora* species can use organic compounds with structures similar to those of diterpenes to synthesize sex hormones. Ojika et al. (2011) proved that A2 strains can use phytol to synthesize α2, whereas A1 strains can use α2 to synthesize α1. In this biosynthetic pathway, four chemical groups on the phytol undergo changes, including the addition of 11-OH and 16-OH to form hormone α2 as well as the formation of a C2–C3 double bond and an α-methyl-branching ketone at C4 on hormone α2 to synthesize hormone α1. Ojika et al. (2011) suggested that cytochrome P450s might be the enzymes catalyzing the modifications that convert phytol to hormone α2. We speculated that alcohol dehydrogenases (ADHs) might contribute to the formation of an α-methyl-branching ketone at C4 on the backbone of hormone α1 via a dehydrogenation after an OH group is added to hormone α2. Additionally, 2-coumarate reductases may help break the C2–C3 double bond on hormone α2 to form hormone α1. We then screened the candidate genes by comparing the changes in their expression levels with the changes in hormone production in the analyzed strains.

**FIGURE 7:**
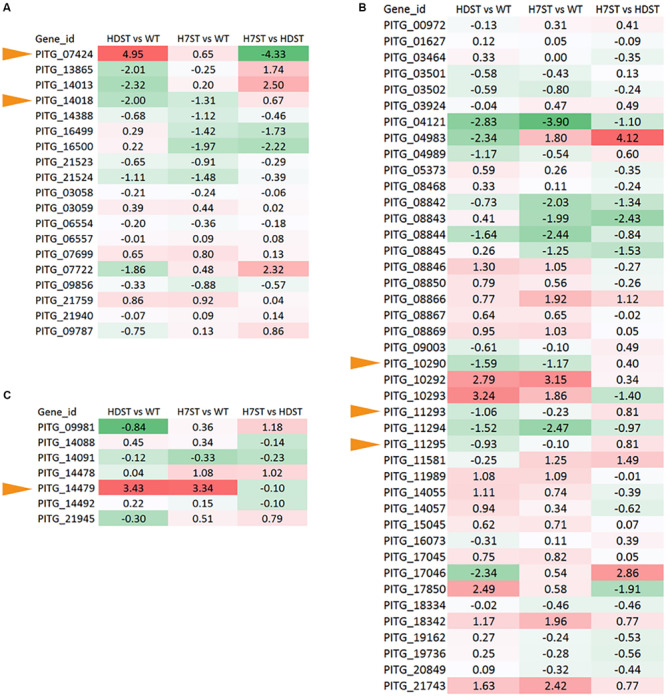
Expression-level differences of genes encoding **(A)** cytochrome P450, **(B)** ADH, and **(C)** 2-coumarate reductase, which alter hormone chemical groups. Red and green denote genes with up- and down-regulated expression levels, respectively. The numbers represent the log_2_(fold-change) values. Yellow arrows represent the hormone synthesis related genes.

We detected 20 P450 genes in the *P. infestans* genome. Because the FPKM of P450 gene *PITG_13866* was less than 1.0, we considered this gene to be unexpressed. The expression patterns of the other P450 genes varied (Figure 7A). The changes in the *PITG_07424* and *PITG_14018* expression levels (*p* < 0.05) were consistent with the hormone production levels in the WT, HDST, and H7ST strains (Figure 1 and Supplementary Figure S2), implying these genes are related to hormone production. The *PITG_07424* expression level was significantly up-regulated in the HDST and H7ST strains, suggesting it may participate in the production of hormone α2. In contrast, *PITG_14018* expression was significantly down-regulated in the HDST and H7ST strains, indicating that it may contribute to the production of hormone α1. Similarly, we identified 47 ADH genes in the *P. infestans* genome, of which 42 were differentially expressed among strains. Three genes with significantly down-regulated expression levels in the HDST and H7ST strains were identified (*PITG_10290*, *PITG_11293*, and *PITG_11295*) (*p* < 0.05, Figure 7B). Moreover, of the seven genes encoding 2-coumarate reductases, the expression of *PITG_14479* was significantly up-regulated in the HDST and H7ST strains (*p* < 0.05, Figure 7C). Accordingly, this gene may mediate the biosynthesis of hormone α1.

### Transcription Factors Related to Hormone Production

**FIGURE 8:**
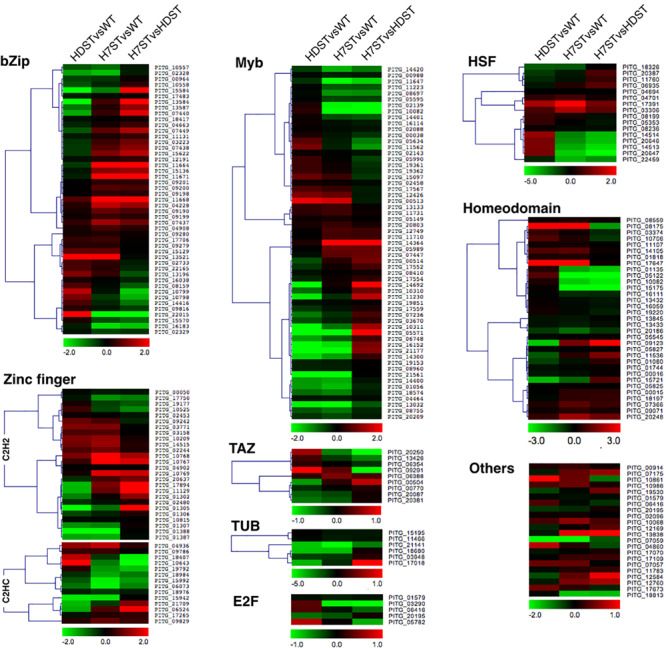
Expression-level differences of transcription factor genes. Red and green denote genes with up- and down-regulated expression levels, respectively. The color bar represents the log_2_(fold-change) value.

Transcription factors are important regulators of gene expression. A thorough analysis of the *P. infestans* genome uncovered 257 transcription factor genes belonging to 18 families (Supplementary Table S7). Our DEG analysis revealed a MYB family gene (*PITG_16152*), a homeodomain family gene (*PITG_08175*), and a CBF/NF-Y family gene (*PITG_10861*) (Figure 8). The *PITG_16152* expression level was significantly down-regulated in the HDST and H7ST strains, whereas the opposite expression trend was observed for *PITG_08175* and *PITG_10861* (*p* < 0.05), suggesting these genes may differentially regulate hormone production.

### Effect of Transcription Factor NF-Y (PITG_10861) on Hormone Production

**FIGURE 9:**
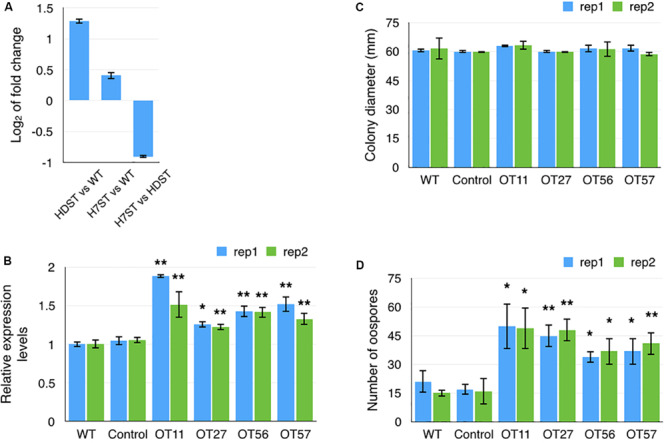
Transcription factor gene *PITG_10861* promotes the production of hormone α2. **(A)** Fold-changes to *PITG_10861* expression levels in the HDST, H7ST, and wild-type strains. Relative expression levels **(B)** and colony diameters **(C)** of wild-type, empty vector control, and four *PITG_10861-*overexpressing transformants. **(D)** Oospore production in the A1 strain induced by the wild-type, empty vector control, and four *PITG_10861-*overexpressing transformants. *p < 0.05; **p < 0.01.

The expression level of a gene (*PITG_10861*) encoding an NF-Y transcription factor, which contains a CBF/NF-Y/archaeal histone domain, was significantly up-regulated in the HDST and H7ST strains (Figure 9A). We speculated that this transcription factor promotes the production of hormone α2. We tested this hypothesis by generating transformants overexpressing *PITG_10861* (OT11, OT27, OT56, and OT57) relative to the corresponding expression levels in the WT and empty vector controls (Figure 9B). The growth rates of the *PITG_10861*-overexpressing transformants were similar to the WT and empty vector control growth rates (Figure 9C). We then quantified the oospore production in the A1 strain induced by these *PITG_10861*-overexpressing transformants as well as the WT and empty vector controls. The data revealed that the A1 strain produced more oospores with OT11, OT27, OT56, and OT57 than with the WT and empty vector controls (Figure 9D). Thus, overexpressing *PITG_10861* increased the production of hormone α2 in *P. infestans*, which is consistent with our hypothesis. Therefore, *PITG_10861* is an important regulator of hormone α2 production.

## Discussion

In this study, we identified many DEGs associated with histone acetylation in *P. infestans* based on Illumina sequencing analyses of a WT *P. infestans* strain, MX5-1, as well as *HDAC* family-silenced and *HDAC7*-silenced transformants. The highly correlated data among the three biological replicates for each strain are indicative of the high reproducibility and reliability of the transcriptome profiling performed in this study. The consistency between the qRT-PCR and RNA-seq data suggests the DEG analysis is accurate. In a previous study, we identified many *P. infestans* genes encoding HATs and HDACs that are expressed in different developmental stages as well as during infections and in response to stresses (Wang et al., 2016). The *HDAC7* expression level is up-regulated during the cyst formation and sexual reproduction stages. Compared with the WT control, the *HDAC*-silenced transformants grew more slowly, with altered hormone production and mating types as well as defective asexual and sexual structures, indicating that HDACs have many biological roles. In this study, we used 6-day-old mycelia as samples for RNA-seq analyses because *Phytophthora* species regularly produce sex hormones during the mycelial growth stage. Thus, the identified DEGs were associated mainly with vegetative growth and the early sexual reproduction stage rather than with infections, the late sexual reproduction stage, or stress responses. To reveal the biological roles of HDACs related to asexual and sexual structures, infections, and stress responses, RNA-seq data for these specific biological stages will need to be generated and analyzed.

The sexual behaviors of *Phytophthora* species are regulated by α hormones (Ko, 1980, 2007; Qi et al., 2005). The three strains examined in this study vary regarding their ability to produce α hormones. By comparing the DEGs between the transformants and the WT control with the changes in hormone production in the transformants, we identified 18 candidate genes related to α hormone synthesis, including three transcription factor genes, two P450 genes, three ADH genes, one 2-coumarate reductase gene, and nine terpene biosynthesis-related genes. We further examined the effect of *PITG_10861* on hormone production by generating transformants in which the gene was overexpressed. Subsequent analyses indicated that overexpressing *PITG_10861* enhanced the production of hormone α2 in *P. infestans*, implying this gene encodes an important regulator of α hormone biosynthesis. This result was also suggestive of the reliability of our analysis. The genetic verification of the regulatory effects of the other genes on α hormone biosynthesis is in progress.

The *PITG_10861* gene encodes the NF-Y transcription factor carrying the CBF/NF-Y/archaeal histone domain. Previous research confirmed that NF-Y is an essential transcription factor for mammalian development, from the early stages to adulthood, and in human pathogenesis (Maity, 2017). In mice, the NF-Y transcription factor interacts with the orphan nuclear receptor steroidogenic factor-1 to regulate the expression of the mouse follicle-stimulating hormone-β gene (*FSH*β) (Jacobs et al., 2003). In *A. thaliana*, NF-YCs interact with the histone deacetylase HDA15 when exposed to light to co-target the promoters of a set of hypocotyl elongation-related genes, thereby modulating the extent of the histone H4 acetylation of the associated chromatin (Tang et al., 2017). Additionally, MYB and homeodomain transcription factors might affect α hormone biosynthesis. Previous studies revealed that MYB transcription factors regulate *P. infestans* sporulation (Xiang and Judelson, 2014). The *AaMYB1* gene and its ortholog *AtMYB61* affect terpene metabolism and trichome development in *Artemisia annua* and *A. thaliana* (Matías-Hernández et al., 2017). We are currently conducting experiments to genetically verify the regulation of α hormone biosynthesis by the MYB and homeodomain transcription factors.

Ojika et al. (2011) reported that A2 isolates can use plant phytols to synthesize the α2 hormone, whereas A1 isolates can use α2 to synthesize α1. In the associated biosynthetic pathway, four phytol chemical groups are altered by the addition of 11-OH and 16-OH to form hormone α2 and the formation of a C2–C3 double bond and an α-methyl-branching ketone at C4 to synthesize hormone α1. Ojika et al. (2011) suspected that oxidizing enzymes (cytochrome P450s) might play a role in the conversion of phytol to hormone α2. In this study, we revealed that P450 genes *PITG_07424* and *PITG_14018* might be involved in sex hormone biosynthesis. The *PITG_07424* expression level was significantly up-regulated in the HDST and H7ST strains, suggesting it may participate in the production of hormone α2. However, *PITG_14018* expression was significantly down-regulated in the HDST and H7ST strains, indicating that it may be involved in the production of hormone α1. In *A. thaliana*, CYP77A6 is an in-chain hydroxylase that functions after CYP86A4, the fatty acid ω-hydroxylase, during the synthesis of 10,16-dihydroxypalmitate, which is required for the synthesis of the cutin polyester at floral surfaces (Li-Beisson et al., 2009). Although the KEGG pathway analysis indicated *PITG_07424* is a homolog of *CYP86*, we were unable to determine which P450 gene is the homolog of *CYP77A6* or *CYP86A4* via a BLAST search because of the low similarity between *P. infestans* and *A. thaliana* P450 genes (Supplementary Figure S5). Consequently, further experimental evidence is needed to elucidate the role of *PITG_07424* related to hormone α2 synthesis. During the conversion of hormone α2 to hormone α1, alcohol dehydrogenases (ADHs) may modify the C4 site to form an α-methyl-branching ketone via a dehydrogenation after the addition of an OH group by P450s. The *PITG_14018* expression profile suggested that this gene might participate in the oxidation during the biosynthesis of α-methyl-branching ketone on sex hormone α1. The genetic verification of the effects of these two cytochrome P450s on α hormone production and their roles in α hormone biosynthesis is in progress.

Considering the diterpene structures of α hormones, we also screened for genes related to terpene synthesis in *Phytophthora* species. Previous studies proved that MAV and MEP pathways are mainly involved in the production of the terpenoid backbone (McGarvey and Croteau, 1995; Rohmer, 1999). However, a genomic examination did not uncover orthologs related to the MEP pathway in *Phytophthora* species. This suggests that only the MAV pathway exists in these oomycetes. After the terpenoid backbone is formed, diterpene compounds can be formed via various pathways, including the pathway involving geranylgeraniol, which is structurally similar to the sex hormones of *Phytophthora* species. However, orthologs of the genes in these pathways have not been detected in *Phytophthora* species. Earlier research indicated that monoterpenes, diterpenes, and tetraterpenes, which are synthesized in the plastids of various organisms, are derived from the isopentenyl diphosphate produced by the MEP pathway (Lichtenthaler, 2000). However, oomycetes lost these plastids during long-term evolution (Tyler et al., 2006; Derelle et al., 2016). The ambiguities related to the terpene biosynthetic pathway are related to these contradictory observations. In the current study, we identified nine terpene biosynthesis-related genes in *P. infestans*. However, their roles in α hormone synthesis remain to be investigated.

This study revealed *P. infestans* genes affected by histone acetylation, including genes related to the biosynthesis of sex hormones and the associated regulation. The data presented herein provide useful inputs for future investigations on the epigenetic mechanisms and the regulation of mating behaviors in *Phytophthora* species.

## Data Availability Statement

All RNA-Seq data generated in this study can be found in PRJNA608542, https://www.ncbi.nlm.nih.gov/bioproject/PRJNA 608542.

## Author Contributions

L-YG and X-WW conceived and designed the experiments and wrote the manuscript. X-WW, J-LL, and Y-RS performed the experiments. X-WW and J-LL analyzed the data. All authors have read and approved the final manuscript.

## Conflict of Interest

The authors declare that the research was conducted in the absence of any commercial or financial relationships that could be construed as a potential conflict of interest.

## Footnotes

1 http://ccb.jhu.edu/software/tophat/index.shtml
