# Supplementary material for: Comparative Transcriptome Analysis Revealed Genes Regulated by Histone Acetylation and Genes Related to Sex Hormone Biosynthesis in Phytophthora infestans
Source: Front Genet. 2020 May 21;11:508. doi: 10.3389/fgene.2020.00508 (PMC7253629; doi:10.3389/fgene.2020.00508)
Supplement: Supplementary file 1 [file Data_Sheet_1.DOCX]

Supplementary Material

Comparative transcriptome analysis revealed genes regulated by histone acetylation and genes related to sexual hormone biosynthesis in Phytophthora infestans

**Xiao-Wen Wang**†**, Jia-Lu Lv**†**, Ya-Ru Shi, Li-Yun Guo***

*** Correspondence:** Li-Yun Guo ppguo@cau.edu.cn, ppguoly@126.com


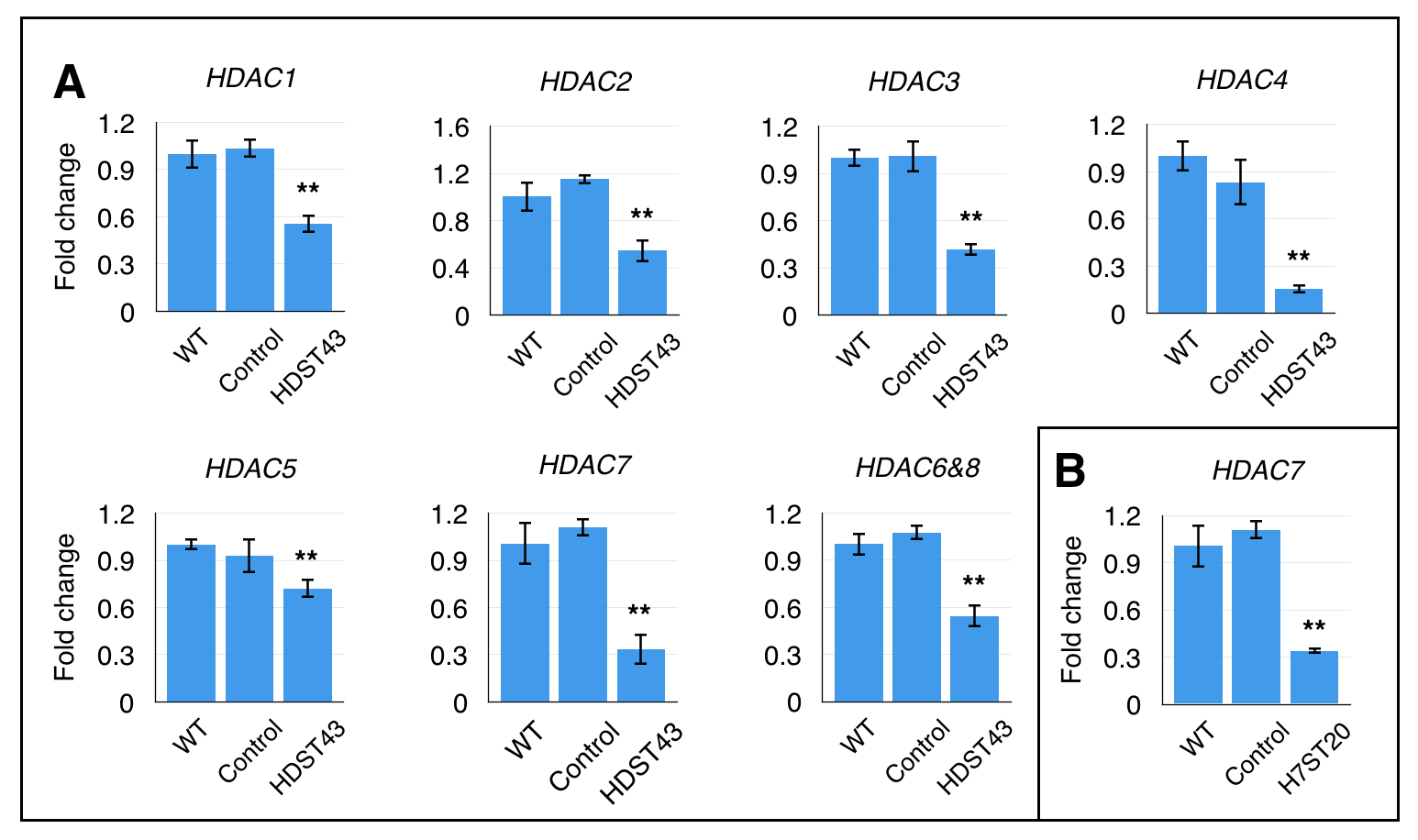


**Supplementary Figure S1.** Relative expression levels of *HDAC* genes in WT, HDST, and H7ST

Fold changes of *HDAC* genes between HDST and WT **(A)**, *HDAC7* between H7ST and WT **(B)** are expressed as the ratio of gene expression after normalization to *EF1*. As extreme high identity between *HDAC6* and *HDAC8*, one pair of primers were used to detect the relative gene expression levels of these two genes. Error bars show s.d. (n=3). Significance was assessed by two-tailed Student’s *t*-test. "*" indicates *p* < 0.05, "**" indicates *p* < 0.01.


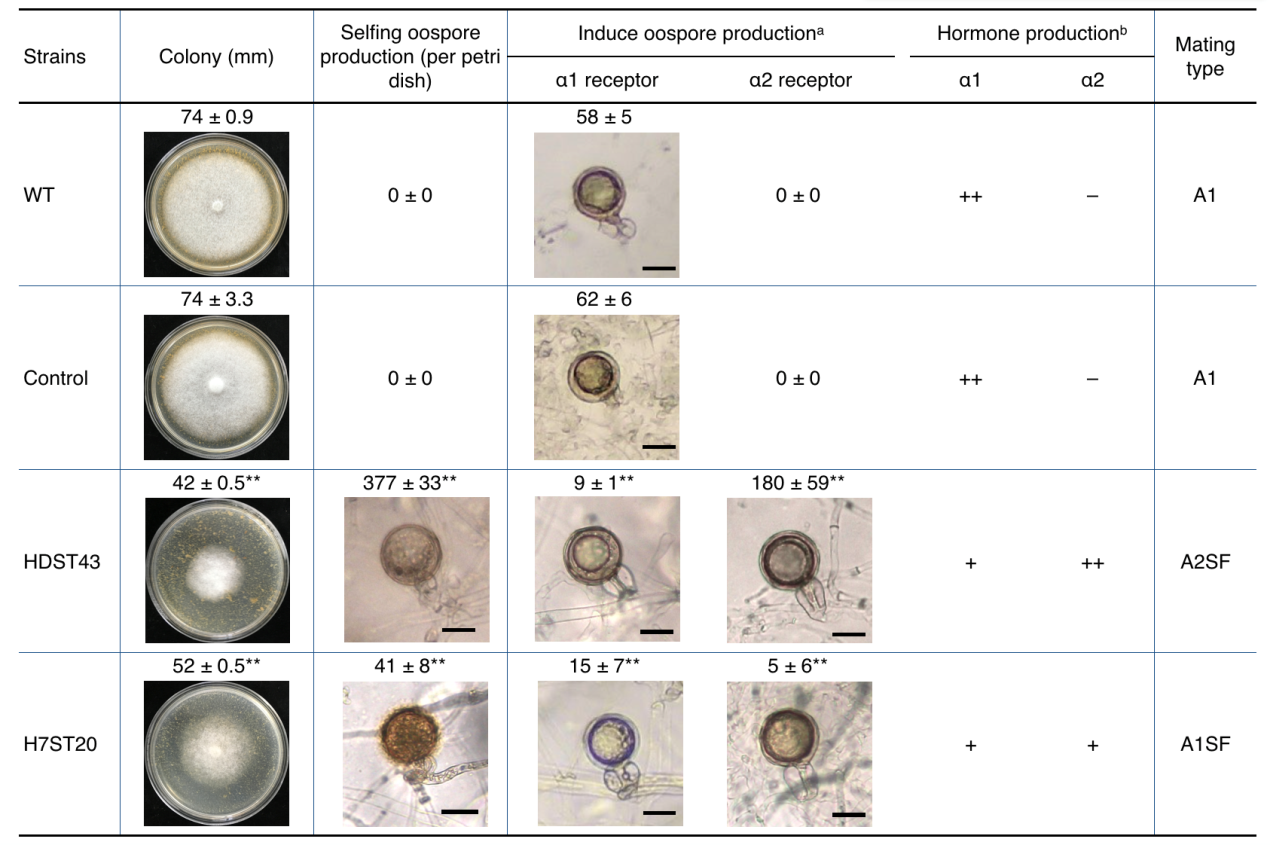


**Supplementary Figure S2.** Phenotype of WT, HDST, and H7ST

^a^For detection of hormone production of transformants, the A1 strain MX5-1 was used as hormone α2 receptor, while the A2SF strain HCL7-7-2 was used as hormone α1 receptor. ^b^Hormone production was determined using the bioassay method. The production of hormone was confirmed if oospores were formed in the receptor strain (or more oospores were formed in receptor strain in comparison with in single strain of HCL7-7-2 when it was used as receptor). “+” means hormone was detected, while “–” means not detected. The amount of oospores formed in receptor indicates the expected relative quantity of hormone produced. The amount of hormone produced was indicated as “+”, “++” and “-”. * and ** indicate the significant difference (**P* < 0.05, ***P* < 0.01) in amount of oospores formed in receptor strains in comparison with its wild type respectively. Significance was assessed by two-tailed Student’s *t*-test.

**
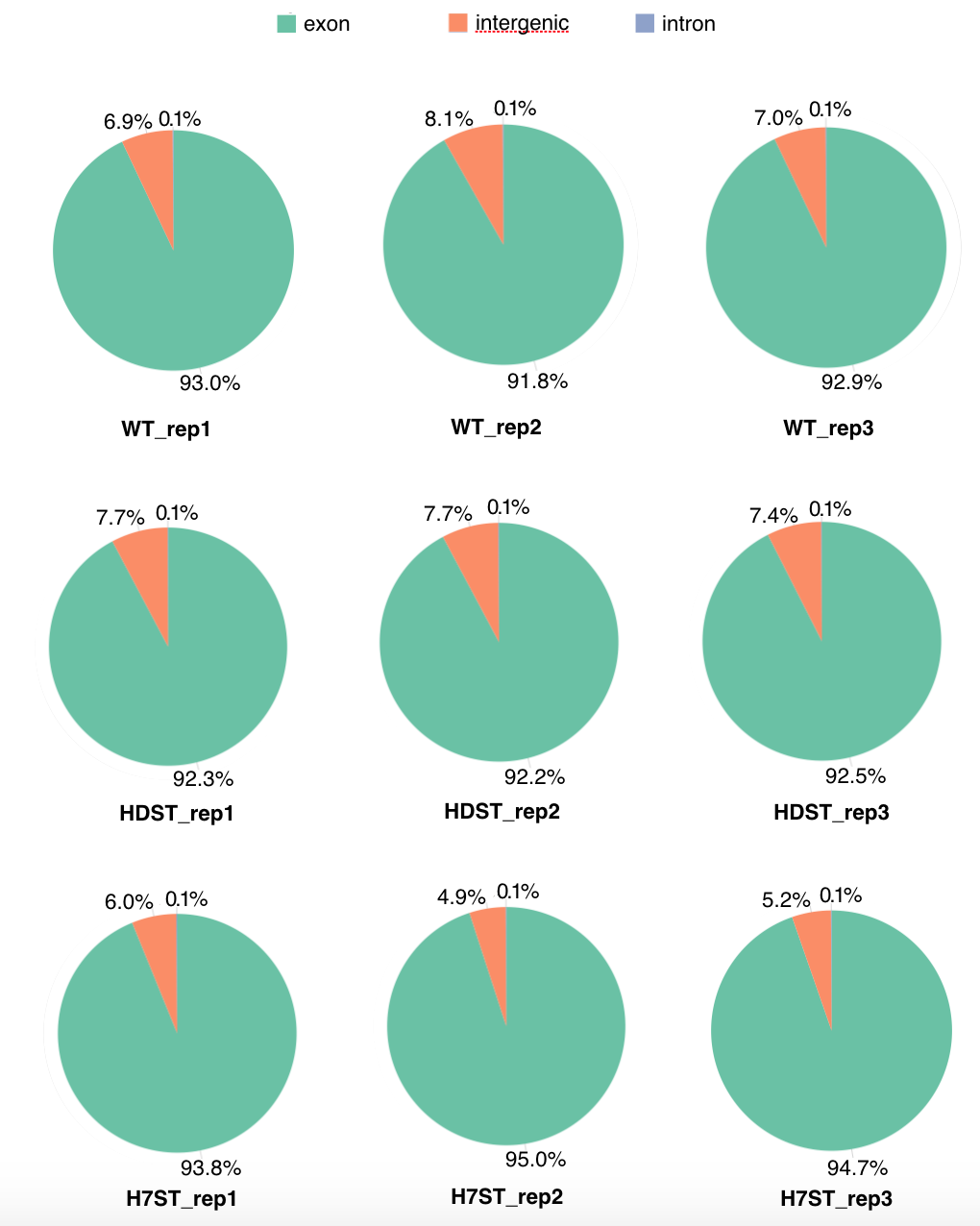
**

**Supplementary Figure S3.** Classification of reads according to mapped region.

Supplement Figure S4. The putative genes and pathways involved in diterpene biosynthesis of *P. infestans*
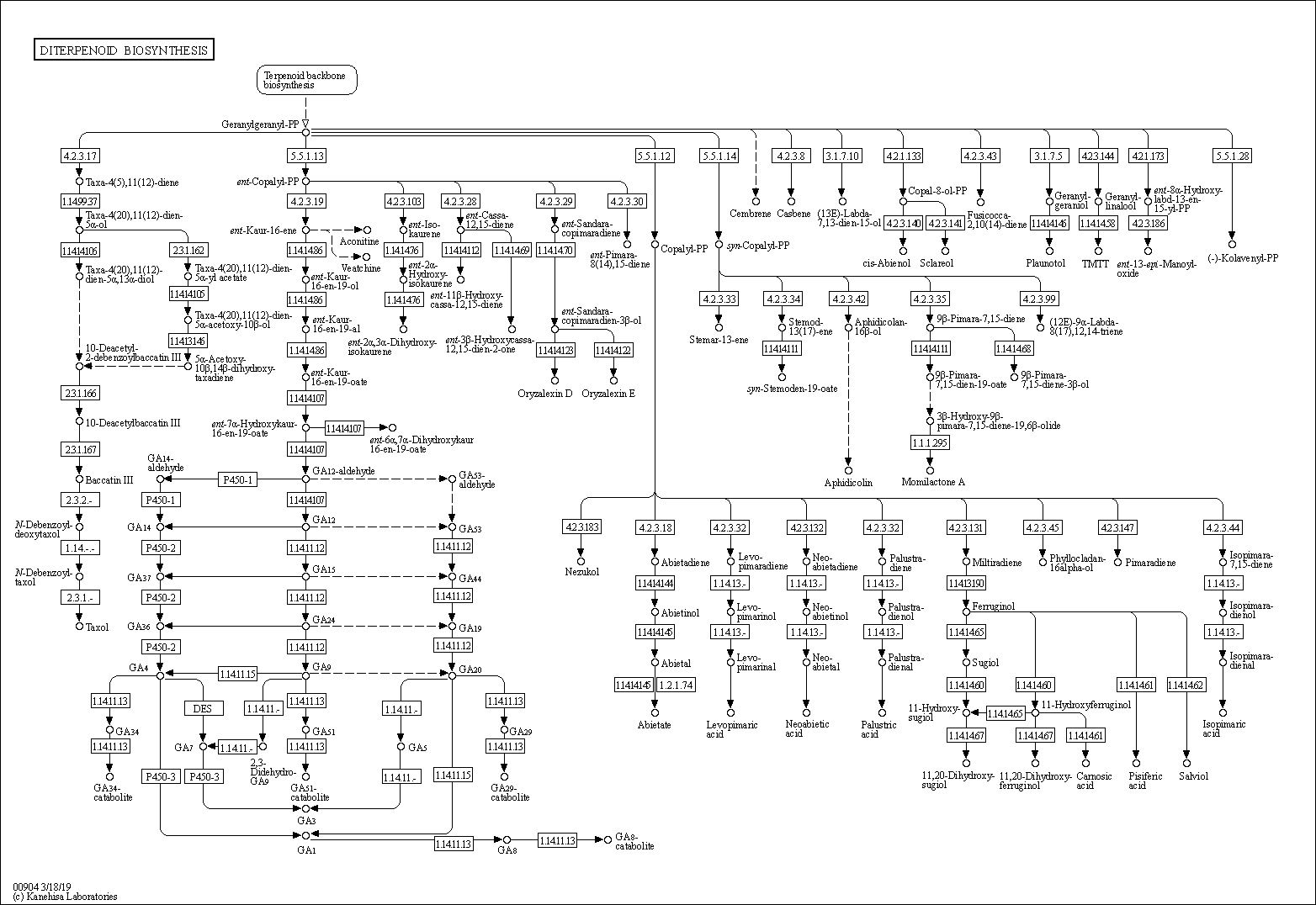

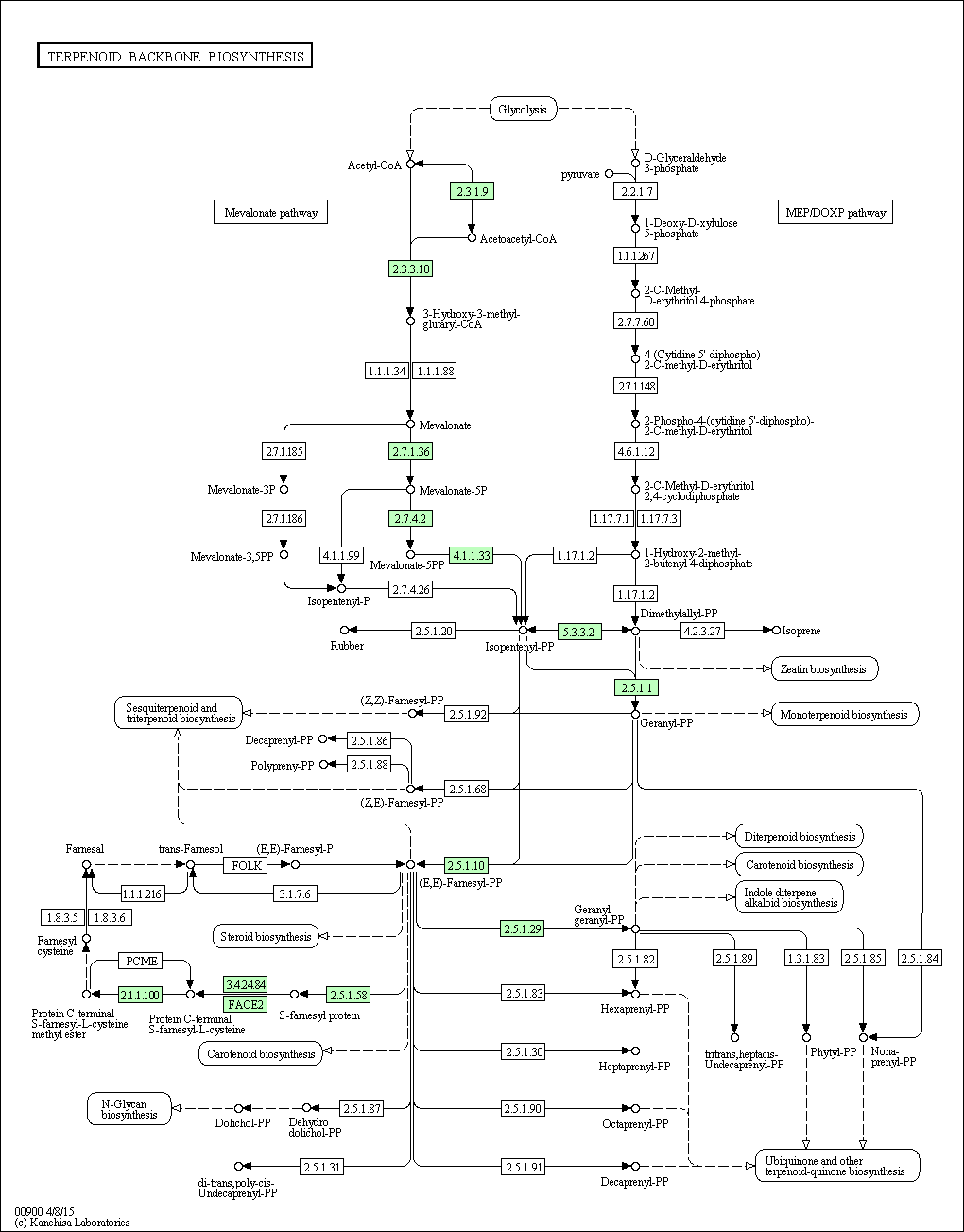


The protein coding genes in green boxes indicate their orthologs existed in *P. infestans*, while the genes in white boxes means no orthologs have been found in *P. infestans*.


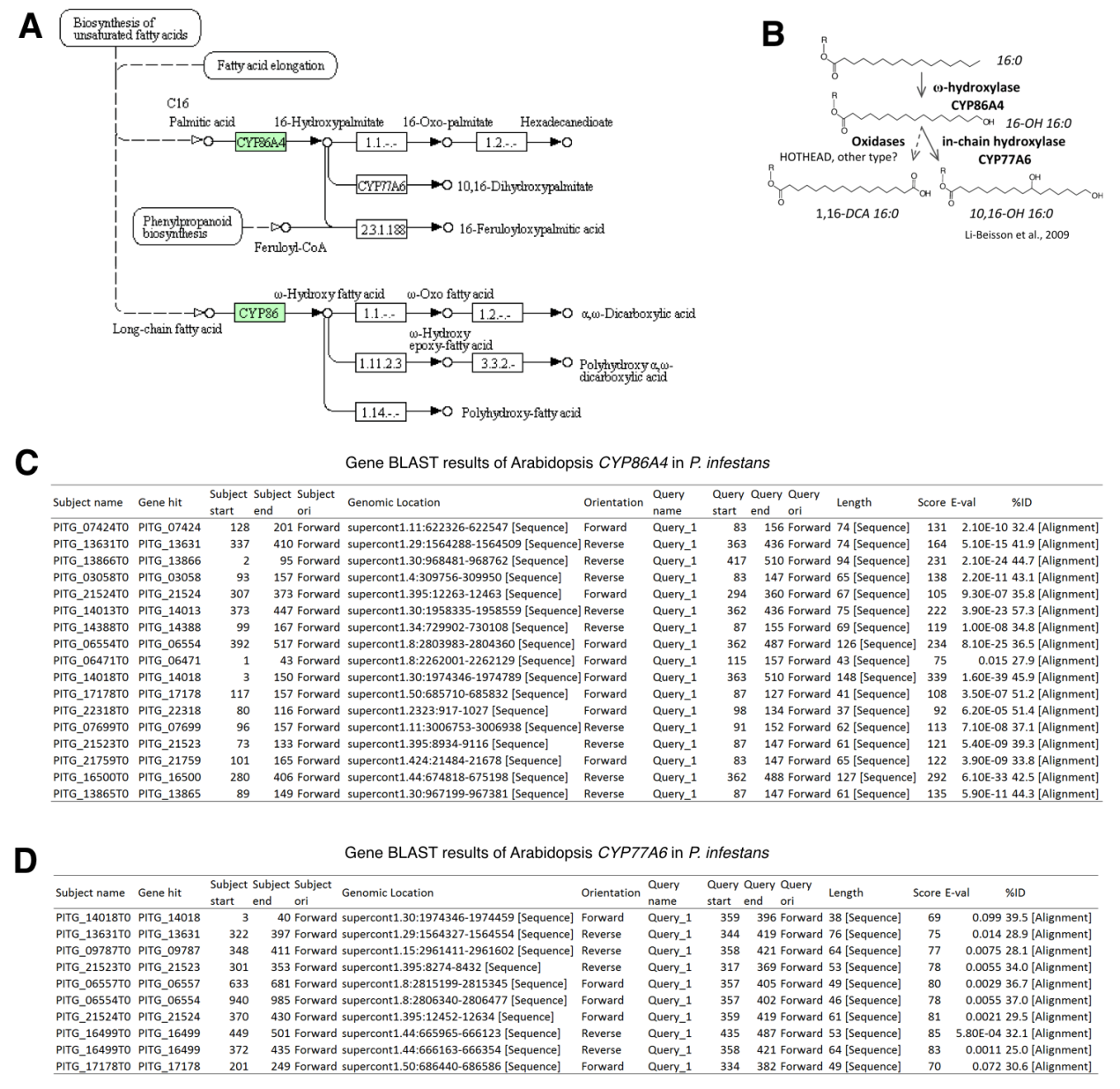


**Supplement Figure S5.** The terpene biosynthesis related CYP450 in *Phytopthora infestans* that was predicted through BLAST search with *Arabdopsis* CYP450 genes

**(A)** P450 genes on biosynthesis pathway of unsaturated fatty acids in *P. infestans*. (B) Functions of CYP86A4 and CYP77A6 in *Arabidopsis*. (C) and (D) Gene BLAST results of Arabidopsis *CYP86A4* and *CYP77A6* in *P. infestans*.

**Supplementary Table S2.** Summary statistics of transcriptome sequencing for three strains of *Phytophthora infestans*.

| **Parameters** | **WT_rep1** | **WT_rep2** | **WT_rep3** | **HDST_rep1** | **HDST_rep2** | **HDST_rep3** | **H7ST_rep1** | **H7ST_rep2** | **H7ST_rep3** |
| --- | --- | --- | --- | --- | --- | --- | --- | --- | --- |
| Total reads | 52737882 | 55830466 | 52301750 | 54633116 | 48512390 | 45800452 | 44382744 | 51465818 | 50542662 |
| Total mapped | 40797483 (77.36%) | 45710804 (81.87%) | 40975383 (78.34%) | 45613438 (83.49%) | 38631077 (79.63%) | 36101715 (78.82%) | 36715654 (82.73%) | 42704120 (82.98%) | 40009349 (79.16%) |
| Multiple mapped | 2465854 (4.68%) | 2785843 (4.99%) | 2147373 (4.11%) | 2769250 (5.07%) | 2232245 (4.6%) | 2210777 (4.83%) | 1909424 (4.3%) | 1977093 (3.84%) | 1883213 (3.73%) |
| Uniquely mapped | 38331629 (72.68%) | 42924961 (76.88%) | 38828010 (74.24%) | 42844188 (78.42%) | 36398832 (75.03%) | 33890938 (74%) | 34806230 (78.42%) | 40727027 (79.13%) | 38126136 (75.43%) |
| Reads map to '+' | 19192526 (36.39%) | 21485903 (38.48%) | 19423425 (37.14%) | 21417619 (39.2%) | 18206694 (37.53%) | 16945237 (37%) | 17417164 (39.24%) | 20384220 (39.61%) | 19086711 (37.76%) |
| Reads map to '-' | 19139103 (36.29%) | 21439058 (38.4%) | 19404585 (37.1%) | 21426569 (39.22%) | 18192138 (37.5%) | 16945701 (37%) | 17389066 (39.18%) | 20342807 (39.53%) | 19039425 (37.67%) |
| Non-splice reads | 33233344 (63.02%) | 37287677 (66.79%) | 33744226 (64.52%) | 36865276 (67.48%) | 31297059 (64.51%) | 29115081 (63.57%) | 29952677 (67.49%) | 35092801 (68.19%) | 32796495 (64.89%) |
| Splice reads | 5098285 (9.67%) | 5637284 (10.1%) | 5083784 (9.72%) | 5978912 (10.94%) | 5101773 (10.52%) | 4775857 (10.43%) | 4853553 (10.94%) | 5634226 (10.95%) | 5329641 (10.54%) |

Total reads：Total number of filtered reads (Clean data).

Total mapped：Total number of reads that can be mapped to the reference genome. In general, this number should be larger than 70% when there is no contamination and the correct reference genome is chosen.

Multiple mapped：Number of reads that can be mapped to multiple sites in the reference genome. This number is usually less than 10% of the total.

Uniquely mapped：Number of reads that can be uniquely mapped to the reference genome.

Reads map to '+'，Reads map to '-'：Number of reads that map to the positive strand (+) or the minus strand (-).

Splice reads：Splice reads can be segmented and mapped to two exons (also named junction reads), whereas non-splice reads can be mapped entirely to a single exon. The ratio of splice reads depends on the insert size used in the RNA-seq experiments.

**Supplementary Table S3.** The number of genes with different expression levels.

| **FPKM**  **Interval** | **WT_rep1** | **WT_rep2** | **WT_rep3** | **HDST_rep1** | **HDST_rep2** | **HDST_rep3** | **H7ST_rep1** | **H7ST_rep2** | **H7ST_rep3** |
| --- | --- | --- | --- | --- | --- | --- | --- | --- | --- |
| 0~1 | 13423  (48.53%) | 13234  (47.85%) | 13614  (49.22%) | 14584  (52.73%) | 14366  (51.94%) | 14434  (52.19%) | 14389  (52.03%) | 14358  (51.91%) | 14190  (51.31%) |
| 1~3 | 1937  (7.00%) | 1926  (6.96%) | 1994  (7.21%) | 1883  (6.81%) | 1831  (6.62%) | 1893  (6.84%) | 1979  (7.16%) | 1981  (7.16%) | 2033  (7.35%) |
| 3~15 | 4508  (16.30%) | 4718  (17.06%) | 4580  (16.56%) | 4697  (16.98%) | 4501  (16.27%) | 4628  (16.73%) | 4493  (16.25%) | 4564  (16.50%) | 4606  (16.65%) |
| 15~60 | 5180  (18.73%) | 5177  (18.72%) | 5019  (18.15%) | 4529  (16.38%) | 4863  (17.58%) | 4693  (16.97%) | 4745  (17.16%) | 4734  (17.12%) | 4746  (17.16%) |
| >60 | 2609  (9.43%) | 2602  (9.41%) | 2450  (8.86%) | 1964  (7.10%) | 2096  (7.58%) | 2009  (7.26%) | 2051  (7.42%) | 2020  (7.30%) | 2082  (7.53%) |

FPKM, short for the expected number of Fragments Per Kilobase of transcript sequence per Millions base pairs sequenced.

The percentages in brackets indicate the ratio between the number of genes with different expression levels and total 27,657 unigenes.
